# Lessons from applied large-scale pooling of 133,816 SARS-CoV-2 RT-PCR tests

**DOI:** 10.1126/scitranslmed.abf2823

**Published:** 2021-02-22

**Authors:** Netta Barak, Roni Ben-Ami, Tal Sido, Amir Perri, Aviad Shtoyer, Mila Rivkin, Tamar Licht, Ayelet Peretz, Judith Magenheim, Irit Fogel, Ayalah Livneh, Yutti Daitch, Esther Oiknine-Djian, Gil Benedek, Yuval Dor, Dana G. Wolf, Moran Yassour, The Hebrew University-Hadassah COVID-19 Diagnosis Team

**Affiliations:** 1 School of Computer Science and Engineering, The Hebrew University of Jerusalem, Jerusalem 91904, Israel.; 2 Department of Developmental Biology and Cancer Research, IMRIC, Faculty of Medicine, The Hebrew University of Jerusalem, Jerusalem 91121, Israel.; 3 Clinical Virology Unit, Hadassah Hebrew University Medical Center, Jerusalem 91120, Israel.; 4 Department of Mathematics, Bar-Ilan University, Ramat-Gan 52900, Israel.; 5 Computing Department of Laboratories and Institutes, Hadassah Hebrew University Medical Center, Jerusalem 91120, Israel.; 6 Department of Medical Neurobiology, Faculty of Medicine, The Hebrew University of Jerusalem, Jerusalem 91121, Israel.; 7 Tissue Typing and Immunogenetics Unit, Hadassah Hebrew University Medical Center, Jerusalem 91121, Israel.; 8 The Lautenberg Centre for Immunology and Cancer Research, IMRIC, Faculty of Medicine, The Hebrew University of Jerusalem, Jerusalem 91121, Israel.; 9 Department of Microbiology and Molecular Genetics, IMRIC, Faculty of Medicine, The Hebrew University of Jerusalem, Jerusalem 91121, Israel.

## Abstract

Pooling multiple swab samples before RNA extraction and real-time reverse transcription polymerase chain reaction (RT-PCR) analysis has been proposed as a strategy to reduce costs and increase throughput of severe acute respiratory syndrome coronavirus 2 (SARS-CoV-2) tests. However, reports on practical large-scale group testing for SARS-CoV-2 have been scant. Key open questions concern reduced sensitivity due to sample dilution, the rate of false positives, the actual efficiency (number of tests saved by pooling), and the impact of infection rate in the population on assay performance. Here, we report an analysis of 133,816 samples collected between April and September 2020 and tested by Dorfman pooling for the presence of SARS-CoV-2. We spared 76% of RNA extraction and RT-PCR tests, despite the frequently changing prevalence (0.5 to 6%). We observed pooling efficiency and sensitivity that exceeded theoretical predictions, which resulted from the nonrandom distribution of positive samples in pools. Overall, our findings support the use of pooling for efficient large-scale SARS-CoV-2 testing.

## Batch testing for SARS-CoV-2

Frequent and accurate RT-PCR–based testing is essential for preventing and managing SARS-CoV-2 infection; however, active infection surveillance is still often limited by time or resources. Cleary *et al.* demonstrate that considering population-level viral prevalence and individual viral loads allows for efficiency gains upon pooled testing with minimal loss of sensitivity, both theoretically and as validated in vitro using human swab and sputum samples. Barak *et al.* show that pooled testing of 133,816 hospital-collected patient nasopharyngeal samples eliminated three quarters of testing reactions with only a minor reduction in sensitivity, demonstrating the efficacy of the approach in the field. Both studies suggest that considered pooling of individual samples before testing could reliably increase SARS-CoV-2 testing throughput.

## Teaser

Adaptive sample pooling for SARS-CoV-2 testing based on positivity rates spares 76% of test reactions without compromising specificity or sensitivity.

## INTRODUCTION

The ongoing coronavirus disease 2019 (COVID-19) pandemic, caused by severe acute respiratory syndrome coronavirus 2 (SARS-CoV-2), has resulted in substantial clinical morbidities and mortality, urging comprehensive virological testing. Major diagnostic challenges have emerged, mainly, the need for high-throughput SARS-CoV-2 reverse transcription polymerase chain reaction (RT-PCR) tests, aimed to detect not only symptomatic but also asymptomatic infectious viral carriers and to screen special or at-risk populations (such as health care personnel or nursing home tenants), to contain viral spread and guide control measures.

These diagnostic challenges together with the consequent shortage in laboratory equipment, reagents, and resources call for the development of a more efficient testing strategy. One promising solution is the application of sample pooling or group testing, a well-developed field in mathematics that allows the identification of carriers in a population of *n* using a number of tests that is smaller than *n*. Group testing can alleviate the supply chain blocks and cut costs while increasing testing throughput. Sample pooling techniques differ in the number and size of pools into which each sample is assigned. In Dorfman pooling (*1*), which is the simplest pooling scheme, each sample is assigned to a single pool, the pools contain equal numbers of samples, and samples are retested individually only if the pool’s test result is positive. In other pooling methods, samples are assigned to multiple overlapping pools to eliminate or at least reduce the number of retested samples (*2*–*5*).

The commonly used diagnostic test for SARS-CoV-2 is based on detection of viral RNA in nasopharyngeal samples by RT-PCR amplification after RNA extraction. Pooling of samples in this context could potentially be used at any stage along the diagnostic workflow, from pooled sample collection to pooled RNA extraction and RT-PCR, or pooled final RT-PCR only (*2*, *6*–*14*), with each approach having pros and cons with regard to test saving versus logistics issues and delays associated with patient and sample retesting.

We and others have recently described the validation and early implementation of sample pooling for SARS-CoV-2 detection (*2*, *6*–*13*, *15*–*17*). In addition, starting July 2020 (*18*), the Food and Drug Administration issued several Emergency Use Authorizations for pooled testing of SARS-CoV-2 and for kits applicable for SARS-CoV-2 pooled testing (*18*, *19*). Most of these studies have used Dorfman pooling (with 4 to 32 samples per pool) and, although largely differing in protocols and stages of pooling used, have suggested sufficient diagnostic accuracy despite an expected loss of sensitivity.

When considering any of the SARS-CoV-2 pooling schemes, there are three crucial concerns: efficiency, or the number of tests spared in practice and how this saving relates to the prevalence rate; sensitivity, or the ability to detect samples with lower viral load of clinical significance despite sample dilution; and operational feasibility, or the technical and logistical implementation of a pooling scheme and its quick adaptation to changes in infection prevalence rates. These concerns cannot be addressed by currently reported studies, which were conducted as a proof of concept, consisting of only hundreds to a few thousands of tested samples examined over a short time period with a relatively constant positive sample rate (usually <1%).

Here, we describe lessons learned from a 5-month period in which we tested 133,816 samples using 17,945 pools. On the basis of early evidence, theoretical considerations, and practical limitations, we chose to implement adaptive Dorfman pooling with pool sizes of five and eight. We evaluated the theoretical and empirical efficiency and sensitivity of our pooling approach, as well as its adaptation to fluctuating rates of positive samples. Overall, we spared 76% of the PCR reactions compared with individual testing, with an acceptable reduction in sensitivity. To our knowledge, this most extensive analysis provides insights into key considerations of efficiency, sensitivity, and feasibility in the actual setting of large-scale sample pooling for SARS-CoV-2 detection.

## RESULTS

**Fig. 1:**
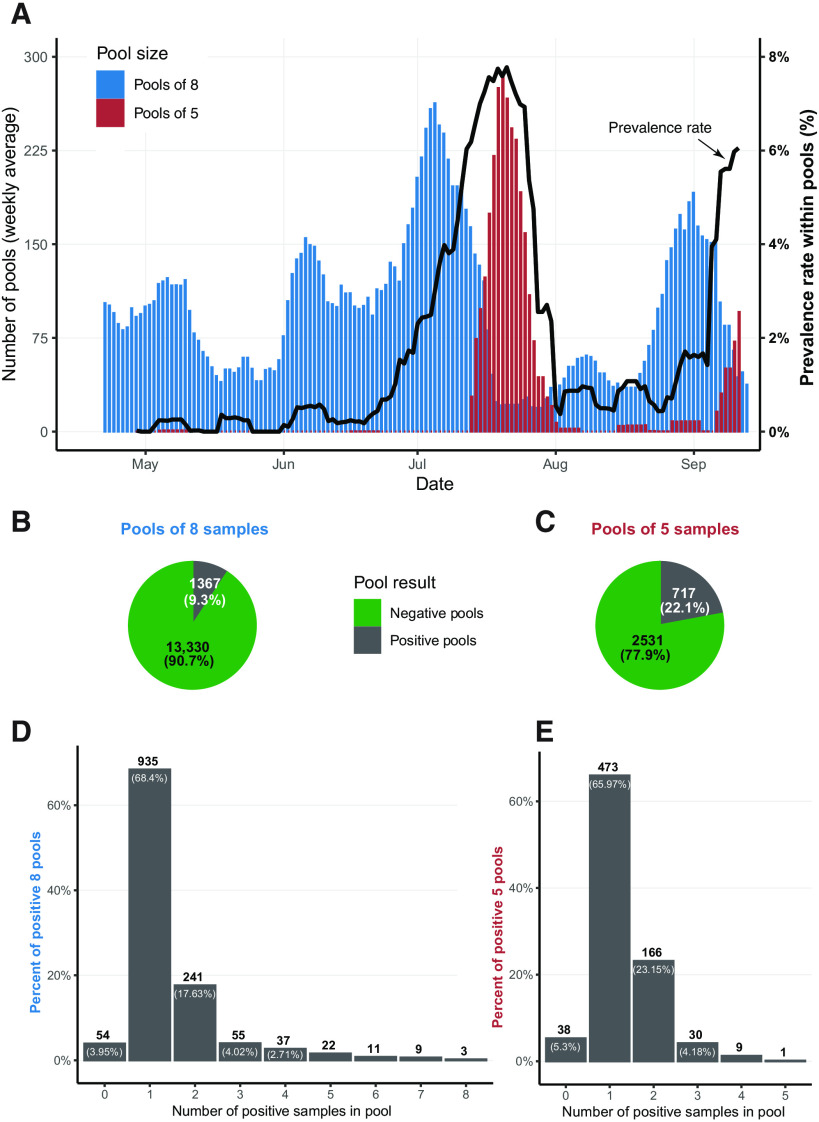
Overall statistics of pool sizes of eight and five. (**A**) Weekly average of eight-sample (blue) and five-sample (red) pools counts, together with the weekly average of the prevalence rate among pooled samples (black). (**B** and **C**) Pool results for eight-sample (B) and five-sample (C) pools, respectively. (**D** and **E**) Counts of positive pools aggregated by the number of positive samples identified within the pool, for eight-sample (D) and five-sample (E) pools.

Between March and mid-September of 2020, we tested 133,816 samples in pools and 121,929 samples using individual tests (nonpooled) at the Hadassah Medical Center in Jerusalem, Israel. One challenge to the pooling scheme stemmed from the fluctuating rates of infection during the pandemic. The infection prevalence rate of pooled samples changed considerably, ranging from a weekly average of 0 to 7.8% [despite the fact that the vast majority (>95%) was obtained from asymptomatic individuals; Fig. 1A], mandating a dynamic adaptation of the pooling scheme. In principle, at low prevalence, using fewer pools of larger pool sizes would lead to a gain in efficiency, as the majority of pools would test negative. However, as prevalence increases, using a larger number of smaller-size pools would be more efficient, as every positive individual would lead to retesting a smaller amount of samples (fig. S1A).

This adaptation of pool size (*n*) according to the prevalence rate (*p*) requires the ability to predict *p* for pooled samples. The prevalence rate in the coming week can be predicted, among other options, according to the prevalence rate in the previous week in pooled or nonpooled samples. The comparison of the weekly average of *p* for pooled and unpooled samples over time suggests that the past week prevalence rate of pooled samples is the better predictor (fig. S1C). Thus, when the prevalence rate in pooled samples increased (from ~1 to ~6%), we switched from eight-sample pooling to five-sample pooling and used a dynamic approach thereafter (alternating the pool size between eight and five) to maintain optimal pooling efficiency (fig. S1, A and B).

In total, we tested 14,697 eight- and 3248 five-sample pools, where 9.3 and 22.1% of the pools tested positive, respectively (Fig. 1, B and C). As all samples in the positive pools were retested individually, we could evaluate the distribution of positive samples within positive pools. Whereas the majority (66 to 68%) of the positive pools contained only one positive sample, 28 to 29% of the positive pools contained two or more positive samples (Fig. 1, D and E). A small number of positive pools (3.9 to 5.3%) did not yield any positive samples when their samples were retested individually. The viral cycle threshold (Ct) values of these pools were usually higher, with a median Ct value of 36.8 and 34.2 for eight- and five-sample positive pools (respectively), whereas all other positive pools had median Ct values of 26.9 and 26.5, respectively. This low percentage of false-positive pools (3.9 to 5.3%) reflects our permissive threshold and the extra caution taken to maintain the sensitivity of pooled sample testing.

A dominant consideration in planning and evaluating the pooling approach is efficiency, defined as the expected number of samples tested using a single RT-PCR reaction. In theory, efficiency is mostly affected by the pool size and the prevalence rate (fig. S1A). We calculated our empirical efficiency (defined as the total number of tested samples divided by the total number of actual RT-PCR reactions performed) as 4.587 and 2.377 for the eight- and five-sample pools, respectively. These values are better than the expected optimal efficiency values for both the eight- and the five-sample pool sizes, under the observed prevalence rates of 1.7 and 5.7%, respectively (Table 1).

**Table 1:** Statistics and efficiency of pool sizes of eight and five. NA, not applicable.

	**Pools of eight**	**Pools of five**	**All together**
Total number of pools	14,697	3248	17,945
Number of positive pools	1367	717	2084
Total number of samples	117,576	16,240	133,816
Number of positive samples	1993	936	2929
Total number of PCR reactions	25,633	6833	32,466
Prevalence rate	1.7%	5.8%	2.19%
Optimal (predicted) Dorfman efficiency	3.9553	2.1891	NA
Our empirical efficiency	4.587	2.377	4.122
*P* value of empirical Dorfman efficiency	<10^−5^	0.00584	NA

As the prevalence of infection changes, so does the pooling efficiency. We observed fluctuations in efficiency values over time, when the empirical efficiency was higher or lower than the theoretical efficiency (fig. S1B). Nevertheless, across time and pool sizes, we performed better than the theoretical efficiency estimations for Dorfman pooling. Overall, we tested 133,816 samples using 32,466 RT-PCR tests with a global efficiency of 4.121, saving 101,350 (76%) RT-PCR reactions.

**Fig. 2:**
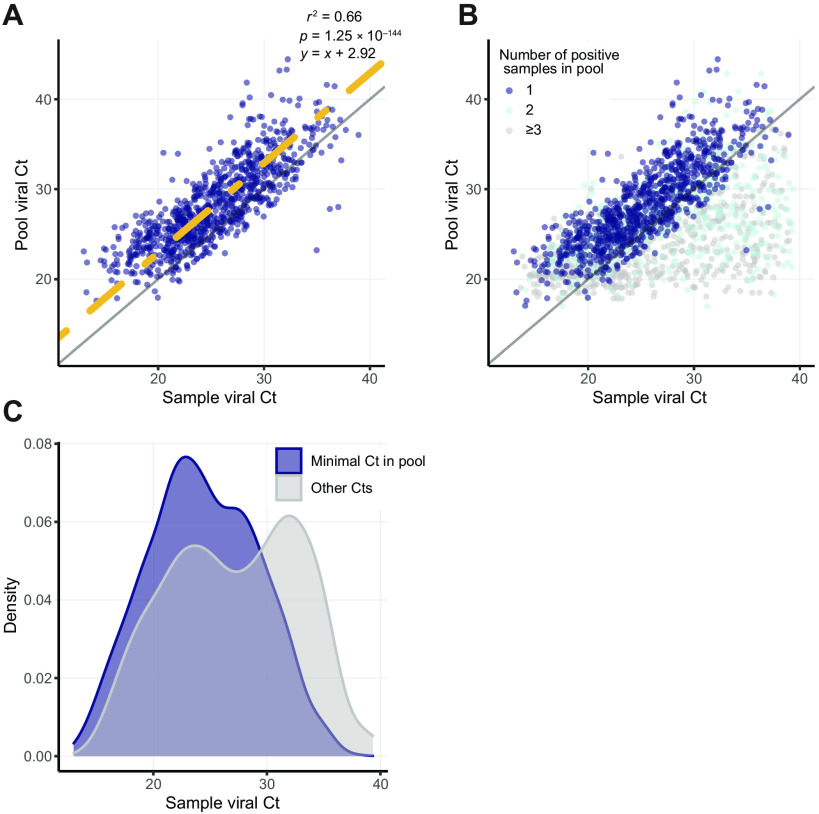
Comparisons of pool Ct and sample Ct. (**A**) Comparison of sample viral Ct (*x* axis) and pool viral Ct (*y* axis) for all 935 amplified eight-sample pools with a single positive sample. Linear regression with a predetermined slope of 1 is marked in yellow, and *y* = *x* is marked in gray. (**B**) As in (A), including also pools with 241 two amplified samples (light blue) and 82 three or more positive samples (gray). (**C**) Distributions of viral Ct values of positive samples in positive pools divided into two groups: samples with the minimal Ct in their pool (blue) and samples with the nonminimal Ct in their pool (gray).

A major concern regarding sample pooling is the expected loss of sensitivity upon sample dilution. We evaluated the sensitivity in our large-scale eight-sample pooling approach, comparing the Ct value of each positive pool with the Ct value of the individually tested positive samples within the pool. Theoretically, an eight-sample pool with a single positive sample should contain only one-eighth of the viral load, which requires three additional PCR cycles (log_2_ of the dilution factor) for detection. Because our PCR assay has a practical limit of sensitivity at 40 cycles, we expect pooling tests to be able to detect samples with viral Ct values up to 37. Individual samples with a Ct value of >37 are expected to be inherent false negatives of the method. To empirically examine the theoretical loss of three Cts in sensitivity, we compared the pool Ct with the individual-sample Ct for 902 pools that contained only a single amplified sample (Fig. 2A). A linear regression analysis of these data revealed a 2.9 Ct increase for the pool (*R*^2^ = 0.66, constraining slope = 1, *P* = 1.25 × 10^−144^; Fig. 2A), in agreement with the theoretical estimation of three Ct elevations. The pooling approach did identify many individual samples that had Ct values of >37 (Fig. 2C). A close examination revealed that these cases were typically found in pools that contained ≥2 samples where the viral gene was amplified and one of the amplified samples had a low Ct value (Fig. 2B). The Ct of a pool is mostly defined by the sample with the highest viral load (lowest Ct) in it; consequently, strongly positive samples lead to individual testing of all samples in the pool, revealing weakly positive “hitchhikers.” The hitchhiker phenomenon explains the better-than-expected sensitivity of our pooling approach. As the average number of positive samples per pool increases, the sensitivity of pooled testing to detect samples with lower viral load (higher Ct) improves (Fig. 2C). This can be caused by either across the board increase in prevalence or by clusters of positive samples that are tested in the same pool.

We have developed a pipeline that consists of guidelines of which samples to pool, hardware to pool the samples (liquid handlers), and software to pool and track the samples for the second stage of examining individual samples within a positive pool. All details regarding this process appear in Materials and Methods, and a video demonstrating the entire process can be found in movie S1.

## DISCUSSION

We used and monitored a large-scale, adaptive eight- and five-sample pooling of nasopharyngeal sample lysates for detection of SARS-CoV-2 over a 5-month period. Data analysis of nearly 135,000 pooled samples revealed high empirical efficiency of sample pooling, outweighting a minor, clinically insignificant loss of sensitivity. Our pooled testing strategy spared 76% of RNA extraction and RT-PCR tests, even in the setting of a changing prevalence rate (<1 to 6%).

Adaptive pooling approaches can maximize resource saving under a fluctuating prevalence rate. The fraction of positive samples tested in pools (*p*) can vary over time due to multiple factors affecting the epidemic kinetics, including changes in public health mitigation measures (for example, social distancing regulations, travel restriction, lockdown, and school closure) (*20*). As a result, the pool size (*n*) required to achieve optimal efficiency shifts. For example, the optimal pool size for *p* = 0.02 (2%) is *n* = 8, but as *p* rises to 0.05 (5%), optimal pool size shrinks to 5 (*1*). Consequently, we tried predicting the positive rate for each week based on the positive rate observed in the previous week, in pooled samples and in nonpooled samples. We found the rate of positives in pooled samples from the previous week to be a better predictor of infection prevalence in pooled samples, probably due to differences between the populations sampled in the two testing methodologies. We therefore adopted a strategy, alternating between pool sizes of eight and five, according to the predicted *p* and the epidemiological information about the source of samples (for example, switching to pools of five when receiving samples from a source highly suspected to have a higher probability of infection). We observed supraoptimal empirical efficiency of pooling, exceeding the predicted efficiency, which could not be explained only by the dynamic switching in pool sizes (see below).

When considering the clinical implementation of group testing, loss of sensitivity is a major concern. The dilution of samples due to pooling may lead to lack of detection in samples with low viral presence (manifested by high Ct values in individual testing). We were not able to estimate the false-negative rate of pooled testing, due to the fact that not all samples included in our analysis were tested individually. However, to estimate the negative predictive value (NPV) of pooled testing, we selected 139 negative pools and retested 1109 samples individually. Only a single sample was found to be positive (Ct = 36.3), suggesting an NPV of 1108/1109 = 99.91%. Our empirical results show a loss of sensitivity as expected based on sample dilution. Given the high sensitivity of current SARS-CoV-2 RT-PCR assays and evidence suggesting lower risk of infectiousness (as measured by cell culture) associated with low presence of viral RNA (high Ct) (*21*–*23*), we believe that the loss of three Cts is a clinically acceptable trade-off when considering the substantial increase in the number of samples tested, as recently suggested (*24*). Our pooling scheme did uncover many samples with high Ct values (>37) that would be expected to be missed in pools, presenting real-life performance that exceeds theoretical expectations, similarly to the observed efficiency trend.

We propose that the better-than-expected performance of pooling in both efficiency and sensitivity aspects is rooted in a single factor: the nonrandom distribution of positive samples in pools. In theory, increased prevalence rates result in decreased efficiency as a common assumption in most models is that samples arrive at random to the diagnostic laboratory. In reality, samples arrive in batches: from colleges, nursing homes, or health care personnel. We sorted samples into pools as they arrived at the laboratory, such that family members and roommates were often pooled together, thereby increasing the number of positive samples within the pool. The presence of multiple positive samples in a single pool can explain both improved efficiency and improved sensitivity. The efficiency improvement is straightforward: A decision to open a positive pool for individual retesting results in the discovery of multiple positive samples with the same number of PCR reactions. The sensitivity improvement is less obvious and stems from the relationship between the sample viral Ct and the pool viral Ct. A single strongly positive sample is sufficient to make the viral load in the pool detected. If the same pool contains additional low viral load samples that would have been otherwise missed upon dilution, these would now “benefit” from the higher viral load samples coexisting in the pool and would be detected when the pool was opened for individual testing. Thus, a nonrandom pool assignment and an increased prevalence rate (which by itself increases the likelihood of having pools with multipositive samples) both contribute to the increased sensitivity. A nonrandom pool assignment together with an adaptive pool size approach further explains our better-than-expected efficiency.

The limitations of this study mostly stem from the retrospective nature of this analysis. These data were not collected for us to study but rather to inform individuals of their infection status. Prospective design that collects information about individuals’ symptoms, demographics and exposure will enable better assignment of samples into pools, achieving higher efficiency. We note that the study reported here was not designed to validate the pooling scheme [which we and others have previously validated (2, 6 to 13, and 15 to 17)] but rather was intended to extract insights from ongoing clinical work, when pooling was used at an unprecedented scale.

One practical implication of our findings is the importance of using preexisting knowledge about incoming samples. Using such information for coassignment of samples suspected to be positive or negative can enable exceeding the theoretical performance of pooling typically calculated under the assumption of random assignment. We encountered considerable logistic hurdles in obtaining a pretest probability for each swab sample but argue that success in such efforts could make pooling work efficient even in settings of very high prevalence.

Last, a common concern with regard to pooling refers to the ease and simplicity of implementation. Although using various pool sizes and performing frequent alternations between them, as well as the use of combinatorial pooling methods in settings of low prevalence rate (2, 4, and 5), may be theoretically more efficient, pooling must be manageable at large scale in a diagnostic laboratory. Combinatorial pooling can be set up efficiently in the laboratory, with predefined pooling schemes that still require a second stage to validate the positive samples (*25*), but not all diagnostic laboratories can handle these complex schemes. We found Dorfman pooling with pool sizes of five or eight both simple and efficient. In addition, we would like to highlight that automation of both sample handling, processing, and result reporting by use of automated liquid handlers and software is crucial for delivering test results quickly and minimizing laboratory errors. We provide a pipeline that consists of guidelines of which samples to pool, hardware to pool the samples (liquid handlers), and software to pool and track the samples for the second stage of examining individual samples within a positive pool.

The long-term containment of COVID-19 will likely involve early identification of outbreaks on the background of low prevalence in the population. Our empirical evidence from testing over 130,000 samples in pools strongly projects on the feasibility and benefits of carefully conducted pooling for surveillance, control, and community reopenings.

## MATERIALS AND METHODS

### Study design

This work is a retrospective analysis of SARS-CoV-2 tests performed by The Hebrew University-Hadassah COVID-19 diagnosis team. From March 2020 to the arbitrary chosen date of 17th of September, 121,929 samples were tested individually, and 139,098 samples were tested using Dorfman pooling. We based the analysis of pooled samples only on pools that showed amplification of the human gene, used as an internal control. In addition, we excluded pools of size different from five or eight and pools missing Ct values due to technical faults. In total, 5282 samples were excluded, and the analysis was performed on 133,816 pooled samples. The study was approved by the Hadassah Medical Center Institutional Review Board with a waiver from the need for informed consent.

### Sample collection

Nasopharyngeal swab samples were collected as they arrived at the Hadassah Medical Center in Jerusalem. The samples were taken at multiple locations in and near Jerusalem and were transferred to the Hadassah Medical Center for evaluation.

### Institutional Review Board

Nasopharyngeal swab samples were collected in 2 ml of viral transport medium (VTM) or directly in the lysis buffer. To inactivate the virus, 220 μl of sample VTM was added to 280 μl of 2× Zymo lysis buffer, followed by 20-min incubation. For the 1:8 pool design, we pooled equal volumes of eight sample lysates to a final volume of 400 μl.

### RNA extraction

RNA was extracted using the QIAsymphony DSP Virus/Pathogen Mini Kit (QIAGEN) on QIAsymphony platform and eluted in 60 μl.

### Reverse transcription polymerase chain reaction

SARS-CoV-2 RNA was detected using multiplex real-time RT-PCR for the simultaneous detection of the SARS-CoV-2–specific *E* gene and a human *ERV3* gene as an internal control (*26*, *27*). Primers and probes were purchased from Integrated DNA Technologies, and the sequences are given in table S1. Real-time RT-PCR was performed using the TaqPath qPCR Master Mix on the QuantStudio 5 Real-Time PCR Instrument (Applied Biosystems Inc.).

The RT-PCR assay, which uses the World Health Organization–approved primers and probes (*26*), was validated on 150 positive and 200 negative nasopharyngeal swab specimens and found to have 100% accuracy with a lower limit of detection of 0.25 copies/μl (corresponding to ~50 copies/ml of clinical sample, with a corresponding detected Ct value of 39). The assay has also been periodically evaluated on external quality assessment/proficiency testing panels [Quality Control for Molecular Diagnostics (QCMD), College of American Pathologists (CAP), Labquality] demonstrating 100% accuracy. All steps that could affect repeatability, reproducibility, sensitivity, specificity, and trueness were evaluated on a regular basis. Further to the initial validation of the pooling method as previously reported (*15*), ongoing evaluation of the pooling across a range of viral loads has been performed by diluting positive nasopharyngeal samples with decreasing viral loads (2000, 1000, 500, 200, 100, and 50 viral copies/ml, corresponding to detected Ct range of 33 to 39) into seven negative samples.

### System support for the pooling process

Unlike individual testing working schemes, pooling requires the ability to efficiently trace all the individual samples associated with a pool. We used a hash file, created automatically by the liquid handling (LiHa) robot. As a batch of 64 individual samples is pooled into eight pools, this file links the eight barcoded individual samples to the corresponding pool barcode. In addition, the date, elution plate barcode, and batch number were automatically added to the file, allowing to quickly locate the individual samples from storage.

To follow a sample from the time it arrives at the laboratory and until a test result is reported, Hadassah Medical Center IT team adapted the Laboratory Information System (LIS) to support pooling and allow dynamic pool size selection. The hash file and the results of the PCR test are integrated into the LIS, automatically reporting negative results for all the samples in a negative pool and assigning all the samples in a positive pool to be retested individually. In addition, laboratory technicians have a wide set of tools enabling efficient and rapid turnaround such as alerts, data analysis tools for the different stages of pooling, and the ability to compare pooling efficiency for different sample sources.

### Pooling pipeline protocol

Our standard operating procedure steps are stated below, illustrated in fig. S2, and a video demonstrating the complete pooling procedure can be found in movie S1.

1) Prepare *n* individual samples barcoded tubes containing 500 μl of mixture of an individual subject VTM + lysis buffer in each tube.

2) Prepare *n*/8 empty tubes with a different set of barcodes. These will later contain the pooled samples.

3) Open and load the individual samples and the empty tubes to the LiHa robot (we used Tecan Freedom Evo 100). In executing pool protocol, first eight individual samples will be pooled to the first pooled sample, and the next eight individual samples will be pooled to the second pooled samples, etc. (50 μl from each, to a total of 400 μl). Alternative faster protocols are available, depending on specifications of the LiHa robot and number of individual samples.

4) Unload the individual samples (now containing 450 μl each), close them with new screw caps, and place them in a tube rack, while maintaining their original order on the LiHa robot’s rack. Store them in a safe and marked box (room temperature/4°C) until pooled samples PCR results are reported.

5) Check that the hash file was created properly and verify that each pooled sample is associated with the correct eight individual samples barcodes.

6) Unload the pooled samples (now containing 400 μl each), close with new screw caps, and transfer to RNA extraction.

7) Perform RNA extraction and RT-PCR on the pooled samples.

8) If the viral gene in the pooled sample is amplified properly (the pooled sample has viral Ct), then locate the relevant individual samples and validate their barcodes using the hash file.

9) Perform RNA extraction and RT-PCR on the suspected individual sample tubes.

### Definition of positive pools

A pool was considered positive if the viral gene was amplified, and individual samples within the pool were retested individually.

### Selection of samples for pooling

By and large, samples from symptomatic and hospitalized patients were tested individually, while samples from screened asymptomatic individuals, such as routinely tested hospital personnel and nursing homes residents and caregivers, were pooled.

### Pooling efficiency

When considering Dorfman pooling, for any given assignment of *p* (prevalence rate) and *n* (pool size), the expected Dorfman optimal efficiency is calculated as ${\left(1+\frac{1}{n}-{\left(1-p\right)}^{n}\right)}^{-1}$, assuming that samples are independent and identically distributed across pools (*1*).

### Pool Ct versus sample Ct calculation

PCR reaction roughly multiplies the amount of the targeted DNA in each cycle of operation. Because of this exponential growth, a pool of size *n* with a single positive sample should have a Ct that is log_2_(*n*) cycles greater than the positive sample’s Ct. For example, when the pool size is 8, this will result in a three-cycle addition.

### Statistical analysis

Calculation of *P* values for empirical efficiency was performed by comparing the empirical efficiency measured to the results of efficiency in 100,000 simulations. For each pool size, five and eight, we used the number of samples and prevalence rate measured for this pool size (Table 1) and simulated (according to the prevalence rate) a positive/negative result for each sample independently. Then, we randomly assigned each sample into a pool and considered a pool to be positive if it included one or more positive samples (assuming no false-negative pools). To conclude the simulation, we calculated the empirical efficiency by dividing the number of samples tested by the number of reactions needed to perform pooled testing. When comparing the pool Ct and the individual sample Ct (Fig. 2A), statistics were calculated using linear regression, forcing a predetermined slope of 1.

## SUPPLEMENTARY MATERIALS

stm.sciencemag.org/cgi/content/full/scitranslmed.abf2823/DC1 Fig. S1. Dorfman efficiency and infection prevalence over time. Fig. S2. Weekly average of percentage of positive samples observed over time, for pooled and unpooled samples. Table S1. Primers and probes used in multiplex RT-PCR. Movie S1. Pooling pipeline protocol. Data file S1. Raw data of all viral and human Ct values in the studied samples and pools.

## Appendix

View/request a protocol for this paper from *Bio-protocol*.
